# The effect of *Asparagopsis taxiformis*, *Ascophyllum nodosum*, and *Fucus vesiculosus* on ruminal methanogenesis and metagenomic functional profiles *in vitro*

**DOI:** 10.1128/spectrum.03942-23

**Published:** 2024-09-30

**Authors:** Timur Yergaliyev, Susanne Künzel, Anna Hanauska, Antonia Rees, Katharina J. Wild, Ásta H. Pétursdóttir, Helga Gunnlaugsdóttir, Christopher K. Reynolds, David J. Humphries, Markus Rodehutscord, Amélia Camarinha-Silva

**Affiliations:** 1 Institute of Animal Science, University of Hohenheim, Stuttgart, Germany; 2 HoLMiR - Hohenheim Center for Livestock Microbiome Research, University of Hohenheim, Stuttgart, Germany; 3 Matís, Reykjavík, Iceland; 4 School of Agriculture, Policy and Development, University of Reading, Reading, United Kingdom; University of Arkansas Fayetteville, Fayetteville, Arkansas, USA

**Keywords:** seaweed, macroalgae, rumen, methanogenesis, Rusitec, Hohenheim Gas Test, microbiome, 16S rRNA gene, metataxonomics, metagenomics

## Abstract

The ruminant-microorganism symbiosis is unique by providing high-quality food from fibrous materials but also contributes to the production of one of the most potent greenhouse gases—methane. Mitigating methanogenesis in ruminants has been a focus of interest in the past decades. One of the promising strategies to combat methane production is the use of feed supplements, such as seaweeds, that might mitigate methanogenesis via microbiome modulation and direct chemical inhibition. We conducted *in vitro* investigations of the effect of three seaweeds (*Ascophyllum nodosum*, *Asparagopsis taxiformis*, and *Fucus vesiculosus*) harvested at different locations (Iceland, Scotland, and Portugal) on methane production. We applied metataxonomics (16S rRNA gene amplicons) and metagenomics (shotgun) methods to uncover the interplay between the microbiome’s taxonomical and functional states, methanogenesis rates, and seaweed supplementations. Methane concentration was reduced by *A. nodosum* and *F. vesiculosus*, both harvested in Scotland and *A. taxiformis*, with the greatest effect of the latter. *A. taxiformis* acted through the reduction of archaea-to-bacteria ratios but not eukaryotes-to-bacteria. Moreover, *A. taxiformis* application was accompanied by shifts in both taxonomic and functional profiles of the microbial communities, decreasing not only archaeal ratios but also abundances of methanogenesis-associated functions. *Methanobrevibacter* “SGMT” (*M. smithii, M. gottschalkii, M. millerae* or *M. thaueri*; high methane yield) to “RO” (*M. ruminantium* and *M. olleyae*; low methane yield) clades ratios were also decreased, indicating that *A. taxiformis* application favored *Methanobrevibacter* species that produce less methane. Most of the functions directly involved in methanogenesis were less abundant, while the abundances of the small subset of functions that participate in methane assimilation were increased.

**IMPORTANCE:**

The application of *A. taxiformis* significantly reduced methane production *in vitro*. We showed that this reduction was linked to changes in microbial function profiles, the decline in the overall archaeal community counts, and shifts in ratios of *Methanobrevibacter* “SGMT” and “RO” clades. *A. nodosum* and *F. vesiculosus*, obtained from Scotland, also decreased methane concentration in the total gas, while the same seaweed species from Iceland did not.

## INTRODUCTION

Ruminants are an important source of meat and dairy products. They also produce methane (CH_4_) (1, 2) through microbial fermentation (3) mainly occurring in the reticulorumen. CH_4_ is known as one of the greatest contributors to greenhouse gas emissions (4). Moreover, CH_4_ production contributes to feed energy loss by the host (5). Numerous efforts have been dedicated to investigating energy loss through methanogenesis in ruminants in the last decades (6, 7). One of the most promising approaches to mitigate CH_4_ production by livestock is the application of specific feedstuffs and feed supplements, including seaweeds (8–10). Such studies were implemented using both *in vivo* (11) and *in vitro* (12–15) experiments. The red seaweed *Asparagopsis taxiformis* is particularly effective in methanogenesis inhibition (13) due to its high bromoform content (16). Bromoform acts as a competitive inhibitor of methanogenesis in virtue of its high chemical similarity to the F_430_ coenzyme (17, 18). However, it has been reported that *A. taxiformis* mitigation of CH_4_ production cannot be explained by only direct competition of bromoform with F_430_ coenzyme and considerably surpasses it (19). The CH_4_ reduction effect of brown seaweeds like *Ascophyllum nodosum* (15, 20) or *Fucus vesiculosus* (21) is proposed to be caused by phlorotannins. However, the effect of these seaweeds on CH_4_ production is not as clear as that of *A. taxiformis*. In addition, tannins have been described to affect the protein metabolism in the rumen. Seaweeds containing tannins may also exert effects on the microbial degradation of dietary proteins (22).

Enteric CH_4_ in ruminants is mostly produced by archaeal methanogens in symbiosis with fiber-degrading bacteria and hydrogen (H_2_) producing protozoa (23, 24). Therefore, CH_4_ reduction may be associated with reduced fiber degradation, an undesirable outcome because the degradation of fiber is a big advantage of ruminants compared to other animals. Although numerous studies have been performed on the effects of seaweeds on rumen microbiome, to the best of our knowledge, there is no study investigating the effect of seaweed additives (particularly, *A. taxiformis*) on microbial functions.

The rumen is an important part of the ruminants’ digestive tract with a very complex microbial community, and therefore, it is difficult to create strictly controlled conditions for *in vivo* studies. Moreover, increased awareness of animals’ welfare stimulates researchers to develop and use *in vitro* alternatives to *in vivo* studies, such as the rumen simulation technique (Rusitec) or Hohenheim gas test (HGT). Rusitec is a semi-continuous cultivation system and allows constant inflow and outflow of the substrates and artificial saliva and, therefore, is well-regulated and balanced (25) The HGT is a widely accepted method for gas production (GP) measurements used for the estimation of digestibility or screening of feed additive effects on methane production (26, 27).

Our objective was to study the effect of five seaweeds on total gas and CH_4_ production, nutrient degradation, and microbial composition and functions in *in vitro* systems. We hypothesized that seaweeds affect methanogenesis not only through biochemical inhibition but also by alteration of microbial (specifically methanogens) composition and functions. Additionally, seaweeds were compared by species and sampling places as two species were harvested at different locations.

## RESULTS

### Experimental design in brief

Five seaweeds as inclusions to the control diet were used to investigate their effect on CH_4_ concentration in the total gas (further referred to as CH_4_ concentration) in two *in vitro* systems, Rusitec and HGT. A total mixed ration (TMR) formulated for cattle was used as a control diet. Five treatments consisted of TMR and the following seaweeds: *A. nodosum* and *F. vesiculosus* harvested in Iceland (AN1 and FV1), the same seaweeds from Scotland (AN2 and FV2), and *A. taxiformis* (AT) from Faial Island, Portugal. For HGT and extended HGT (eHGT), all seaweeds were used at the inclusion level 5% to TMR based on a dry matter (DM). For the Rusitec, the seaweed inclusion level was 2.5% for all treatments. The rumen content for the *in vitro* systems was obtained from rumen-cannulated cows.

Microbiota analyses were performed only for the Rusitec experiment. In order to obtain a better understanding of the seaweed effect on microbiota composition, samples were taken from the initial rumen solid phase (RSP) and rumen fluid (RF) and from the Rusitec feed residues (FRs) and fermenter liquid (FL).

### Gas and methane production

**Fig 1:**
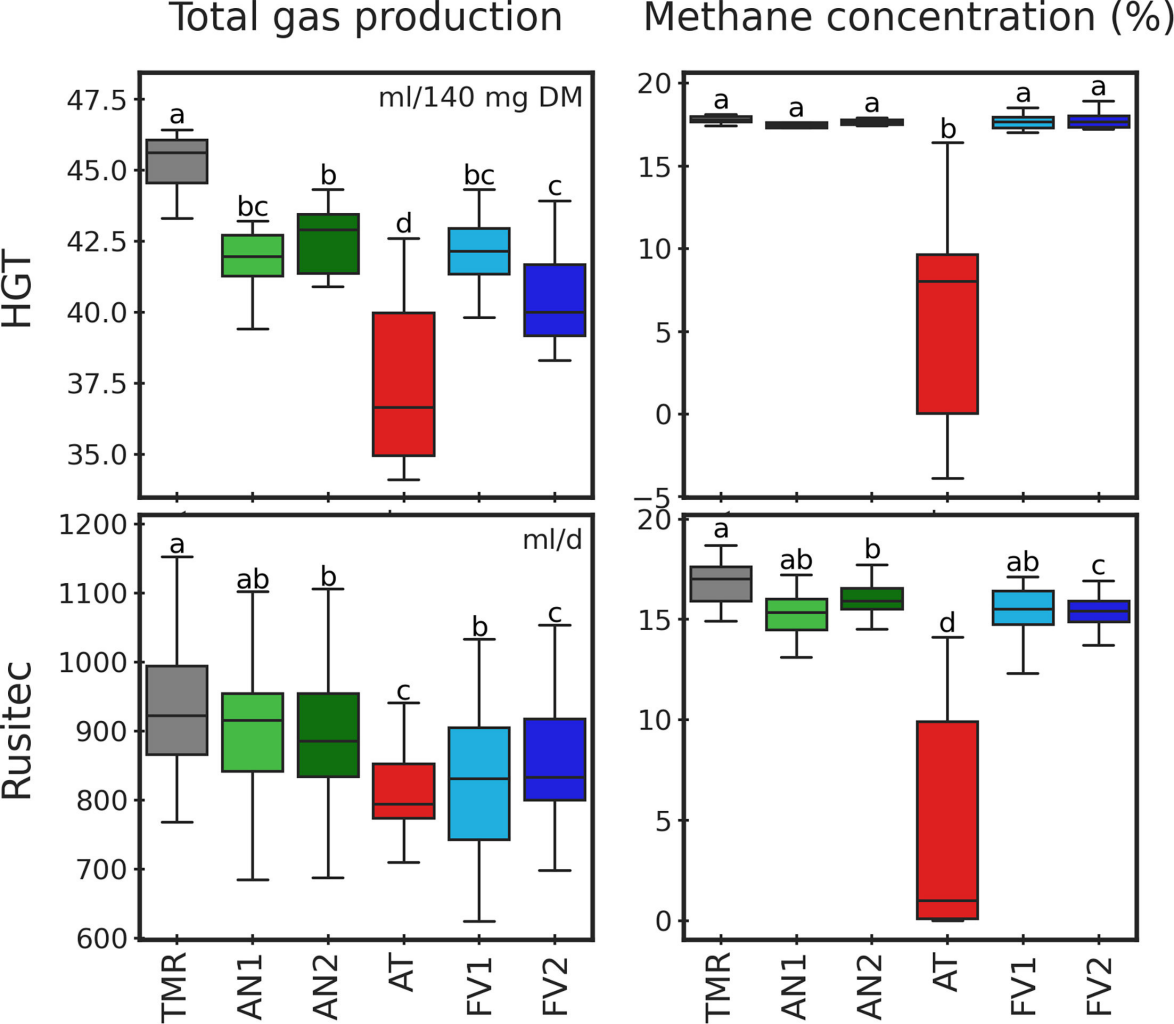
Total gas production and methane concentration in HGT and Rusitec experiments. For total gas production, units are indicated at the upper right part of the corresponding subplot.

The CH_4_ concentration determined in the HGT decreased by the supplementation of AT compared to TMR (*P* = 0.007). All seaweeds decreased the GP compared to TMR treatment in the HGT (*P* < 0.001; Fig. 1; Table S1). In Rusitec, the supplementation of AN2, FV2, and AT resulted in lower GP and CH_4_ concentrations than TMR alone (both *P* < 0.001; Fig. 1; Table S2). The greatest reduction in CH_4_ concentration compared to TMR alone was caused in both experiments by the supplementation of AT (reduction of 11.9% points in the HGT and 12.4% points in the Rusitec). Regarding GP, the lowest values were observed for treatment FV2 in the Rusitec and treatment AT in the HGT.

### Metabolizable energy and nutrient degradation

The metabolizable energy (ME) estimated with the HGT was reduced by the supplementation of AN1 and FV1 compared to TMR alone by 0.1 MJ/kg DM, respectively (*P* = 0.008; Table 1). In the Rusitec, both FV treatments decreased the degradation of all analyzed nutrients (*P ≤* 0.001; Table 1). Only the crude protein (CP) degradation was lower for AT and the AN2 than for the TMR treatment. The AN1 had a lower CP and acid detergent fiber on an ash-free basis (ADFom) degradation than the TMR treatment.

**TABLE 1:** Nutrient degradation in the feed bags of the six experimental treatments in the Rusitec (d 7–12, *n* = 4) and metabolizable energy observed in the HGT (*n* = 8)^*a*^

	DM%	OM%	CP%	ADFom%	aNDFom%	MEMJ/kg DM
TMR	39.8^a^	40.0^a^	35.5^a^	28.0^a^	21.9^a^	12.3^a^
AN1	38.4^ab^	38.5^ab^	31.0^b^	24.8^b^	20.3^ab^	12.2^b^
AN2	38.0^ab^	38.1^ab^	31.2^b^	27.5^a^	20.3^ab^	12.3^ab^
AT	39.4^a^	39.3^a^	31.6^b^	26.6^ab^	22.4^a^	12.0^ab^
FV1	36.6^bc^	36.7^bc^	28.4^c^	24.7^b^	18.0^bc^	12.2^b^
FV2	36.2^c^	36.2^c^	27.2^c^	21.0^c^	16.9^c^	12.3^ab^
*Pooled SEM*	0.68	0.68	0.80	1.12	0.94	0.05
*P*	0.001	<0.001	<0.001	<0.001	<0.001	0.008

^*a*^ AN, Ascophyllum nodosum; AT, Asparagopsis taxiformis; FV, Fucus vesiculosus, used with 2.5 % inclusion level; DM, Dry matter; OM, Organic matter; CP, Crude protein; ADFom, Acid detergent fiber on ash free basis; aNDFom, Neutral detergent fiber on ash free basis; ME, Metabolizable energy. Within a column, entries without a common superscript differ (*P* < 0.05).

Effective “utilizable crude protein at the duodenum” (uCP) in the eHGT was not affected by seaweed supplementation at an assumed passage rate of 2%/h, and rumen undegradable protein (RUP) was also not affected at the assumed passage rates of 2%/h and 5%/h (Table 2). At an assumed passage rate of 8%/h, effective RUP was significantly higher by 9% (AN2) to 12% (FV1) of CP in treatments AN2, AT, and FV1 than in TMR alone (*P* = 0.014). All seaweeds increased the effective uCP compared to TMR at an assumed passage rate of 8%/h by 8 (AT) to 13 (FV1) g/kg DM (*P* = 0.001). At an assumed passage rate of 5%/h, both AN treatments and FV1 increased effective uCP compared to TMR alone by 16 (AN2) to 20 (FV1) g/kg DM (*P* = 0.015).

**TABLE 2:** Effective utilizable crude protein at the duodenum (uCP, g/kg DM) and ruminally undegradable crude protein (RUP, % of CP) for different assumed ruminal passage rates in the eHGT^*a*^

	Effective uCP			Effective RUP		
	g/kg DM			% of CP		
Passage rate	8%/h	5%/h	2%/h	8%/h	5%/h	2%/h
AN1	210^a^	189^a^	148	48^ab^	34	5.5
AN2	208^a^	188^a^	150	51^a^	45	31
AT	206^a^	182^ab^	134	53^a^	40	14
FV1	211^a^	192^a^	154	54^a^	46	29
FV2	207^a^	182^ab^	134	49^ab^	37	15
TMR	198^b^	172^b^	122	42^b^	32	12
*Pooled SEM*	*5.04*	*3.05*	*10*	*2.5*	*4.0*	*8.0*
*P*	0.001	0.015	0.072	0.014	0.112	0.197

^*a*^ AN, Ascophyllum nodosum; AT, Asparagopsis taxiformis; FV, Fucus vesiculosus; TMR, total mixed ration; DM, dry matter; CP, crude protein.^.^ Within a column, entries without a common superscript differ (*P* < 0.05).

### Fermentation characteristics

The pH (6.96; *P* = 0.101), redox potential (−312 mV; *P* = 0.064), and temperature (39.3°C; *P* = 0.102) measured in the fermenters did not differ among the treatments. Supplementation of AT decreased the production of NH_3_-N, acetate, isobutyrate, butyrate, and the acetate to propionate ratio (C2:C3) but increased the production of iso-valerate and valerate compared to TMR alone (*P ≤* 0.001; Table 3). The other seaweeds decreased the production of NH_3_-N and all volatile fatty acids (VFAs) compared to TMR alone, except acetate and propionate for AN1, iso-valerate for AN2 and FV2, and C2:C3 for all.

**TABLE 3:** NH_3_-N and VFA production analyzed in the effluent of the six experimental treatments in the Rusitec (d 7–13, *n* = 4)^*a*^

	NH_3_-N mmol/d	Acetate mmol/d	Propionate mmol/d	Iso- butyrate mmol/d	Butyrate mmol/d	Iso-valerate mmol/d	Valerate mmol/d	VFA_total_ mmol/d	C2:C3
TMR	9.00^a^	16.7^a^	8.97^ab^	0.42^a^	4.24^a^	0.95^b^	2.65^b^	33.9^a^	1.86^ab^
AN1	7.41^b^	16.4^a^	8.79^bc^	0.37^b^	3.65^c^	0.77^c^	2.55^c^	32.5^b^	1.87^ab^
AN2	7.32^bc^	15.8^b^	8.33^e^	0.36^b^	3.81^b^	0.91^b^	2.49^d^	31.7^c^	1.91^a^
AT	7.22^cd^	14.2^d^	9.08^a^	0.34^c^	3.60^cd^	2.76^a^	2.90^a^	32.9^b^	1.56^c^
FV1	7.03^d^	15.8^b^	8.57^cd^	0.34^c^	3.49^d^	0.60^d^	2.47^d^	31.3^c^	1.83^ab^
FV2	6.73^e^	14.8^c^	8.48^de^	0.32^d^	3.22^e^	1.00^b^	2.52^cd^	30.4^d^	1.76^b^
*Pooled SEM*	*0.185*	*0.482*	*0.192*	*0.009*	*0.085*	*0.097*	*0.041*	*0.70*	*0.061*
*P*	<0.001	<0.001	<0.001	<0.001	<0.001	<0.001	<0.001	<0.001	0.001

^*a*^ AN, Ascophyllum nodosum; AT, Asparagopsis taxiformis; FV, Fucus vesiculosus, used with 2.5 % inclusion level. Within a column, entries without a common superscript differ (*P* < 0.05).

### Metataxonomics

After demultiplexing, denoising, chimeras removing, and all filtering steps of 16S rRNA gene amplicons 135 archaeal (with a total frequency 3,648,624) and 5,961 bacterial (total frequency 6,948,793) amplicon sequence variants (ASVs) retained.

#### ASVs diversity and composition

**Fig 2:**
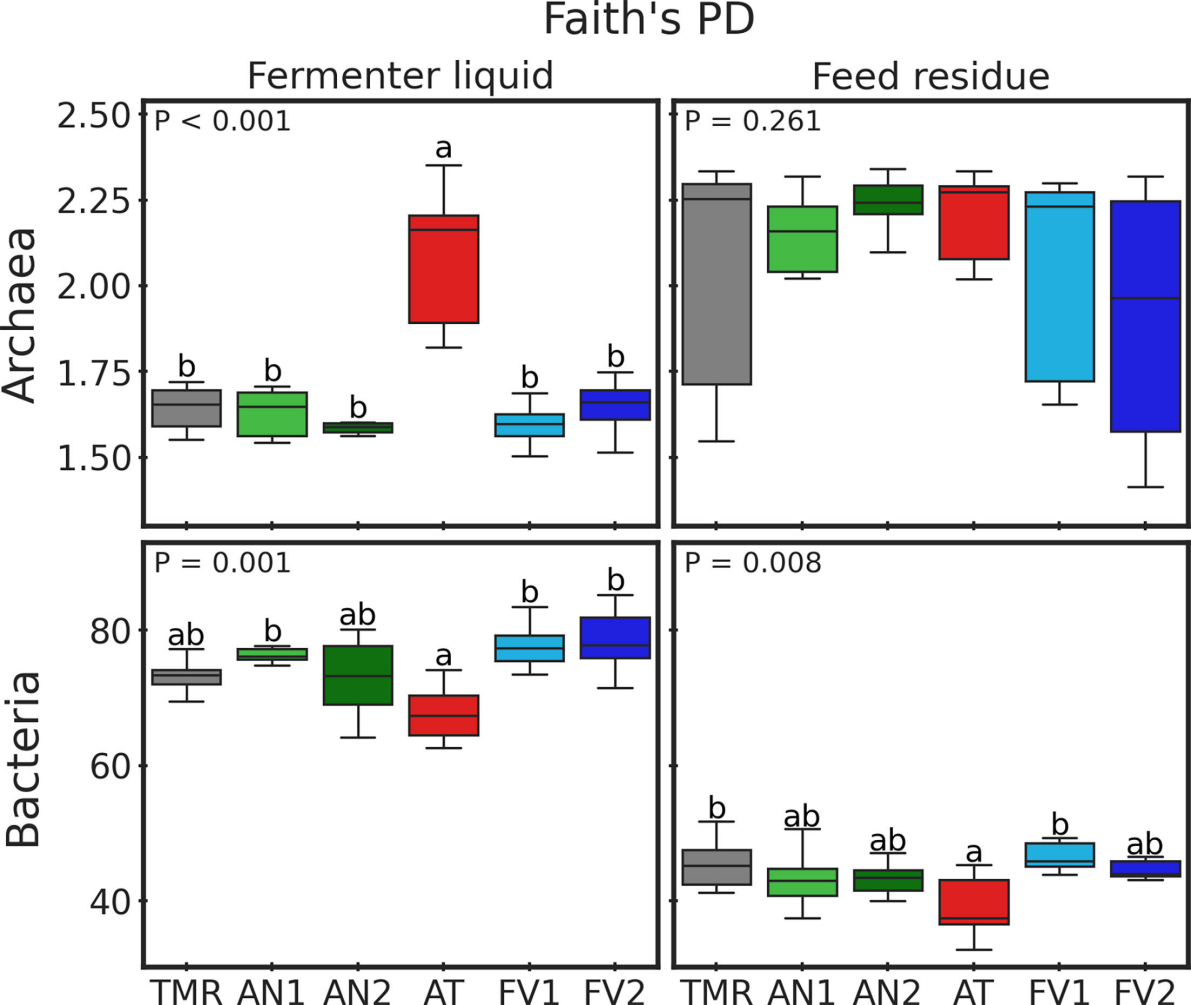
Effect of seaweed supplementation on archaeal and bacterial Faith’s PD. Samples are grouped by sample type and treatment. *P*-value of general ANOVA analysis of variance (ANOVA) test plotted in the upper left part of subplots. Significance of pairwise *t* tests denoted by letters and based on adjusted *P*-values.

Alpha diversity of both bacterial and archaeal communities was assessed by Faith’s phylogenetic diversity (PD; Fig. 2; Fig. S1). Archaeal Faith’s PD was affected by seaweed supplementation only in FL (*P* < 0.001). Pairwise *t* tests indicated that AT had greater diversity compared to all other treatments (all *P-adj* <0.001). Regarding bacterial reads, the effect of seaweed supplementation was detected in both FL (*P* = 0.001) and FRs (*P* = 0.008) sample types. In FL, AT had lower diversity compared to treatments AN1, FV1, and FV2 (*P-adj* ≤ 0.012), and in FR, AT had Faith’s PD lower than TMR and FV1 (*P-adj* = 0.006).

**Fig 3:**
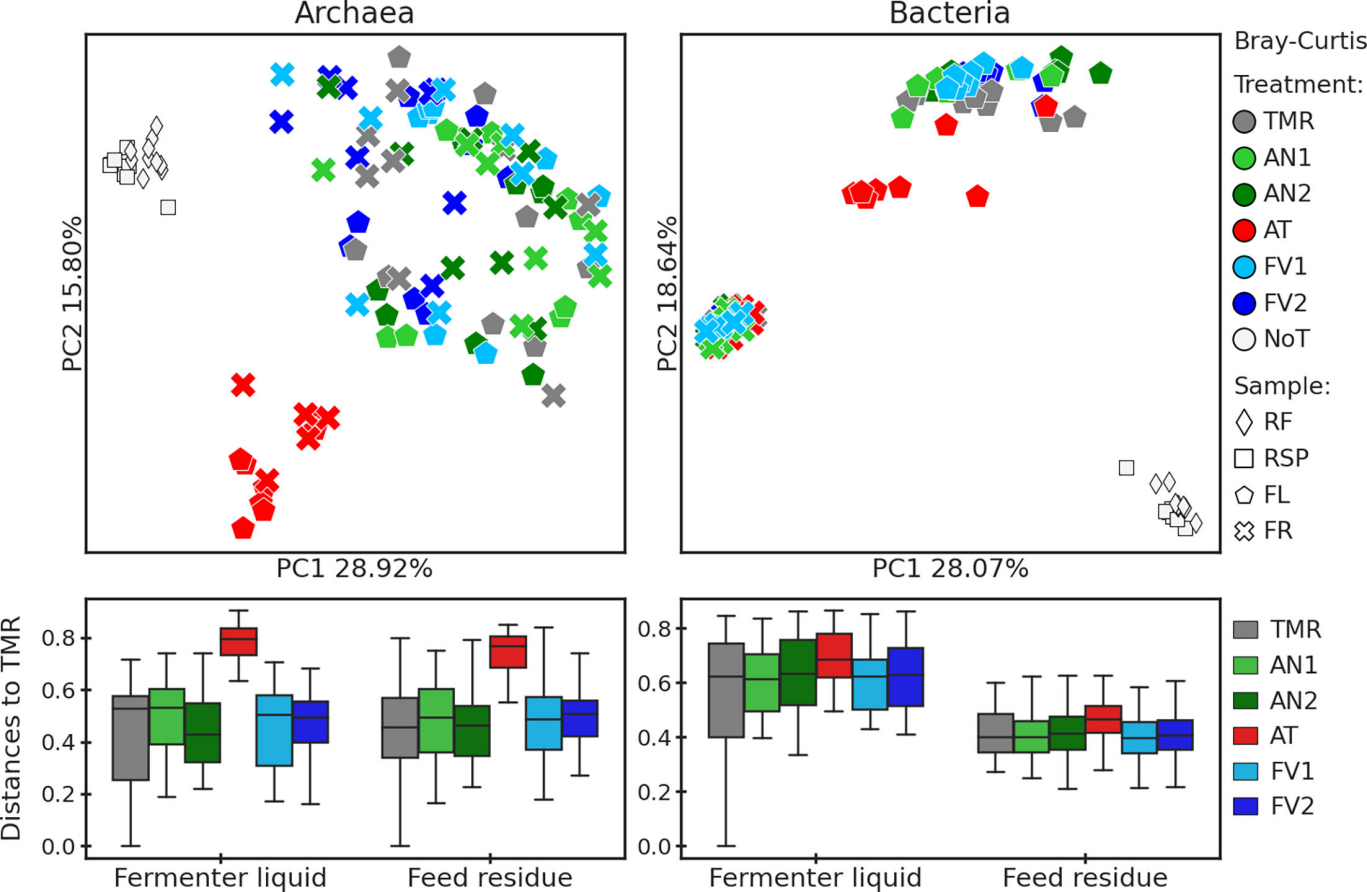
Principal coordinate analysis (PCoA) plots and distances to the TMR based on the archaeal and bacterial Bray-Curtis matrices. Treatments are denoted by the color, sampling days by the size, and sample types by the shape. “NoT” stands for “no treatment.”

To measure beta diversity, Bray-Curtis distances were calculated and plotted as principal coordinate analysis (PCoA) and distances to the TMR (Fig. 3). Seaweed supplementation had a significant impact in all sample types with treatment for both archaea and bacteria (all *P* = 0.001). When compared pairwise, AT was different from all other treatments both in archaeal and bacterial datasets (*P-adj* ≤ 0.006) and the most distant treatment from the TMR alone. Regarding other treatments, in archaeal FR samples, FV2 was different from TMR, AN1, and AN2 (*P-adj* ≤ 0.015). In the bacterial dataset, FV1 was significantly distant from TMR and AN2 (*P-adj* ≤ 0.035) in FL.

#### Genera relative abundances

**Fig 4:**
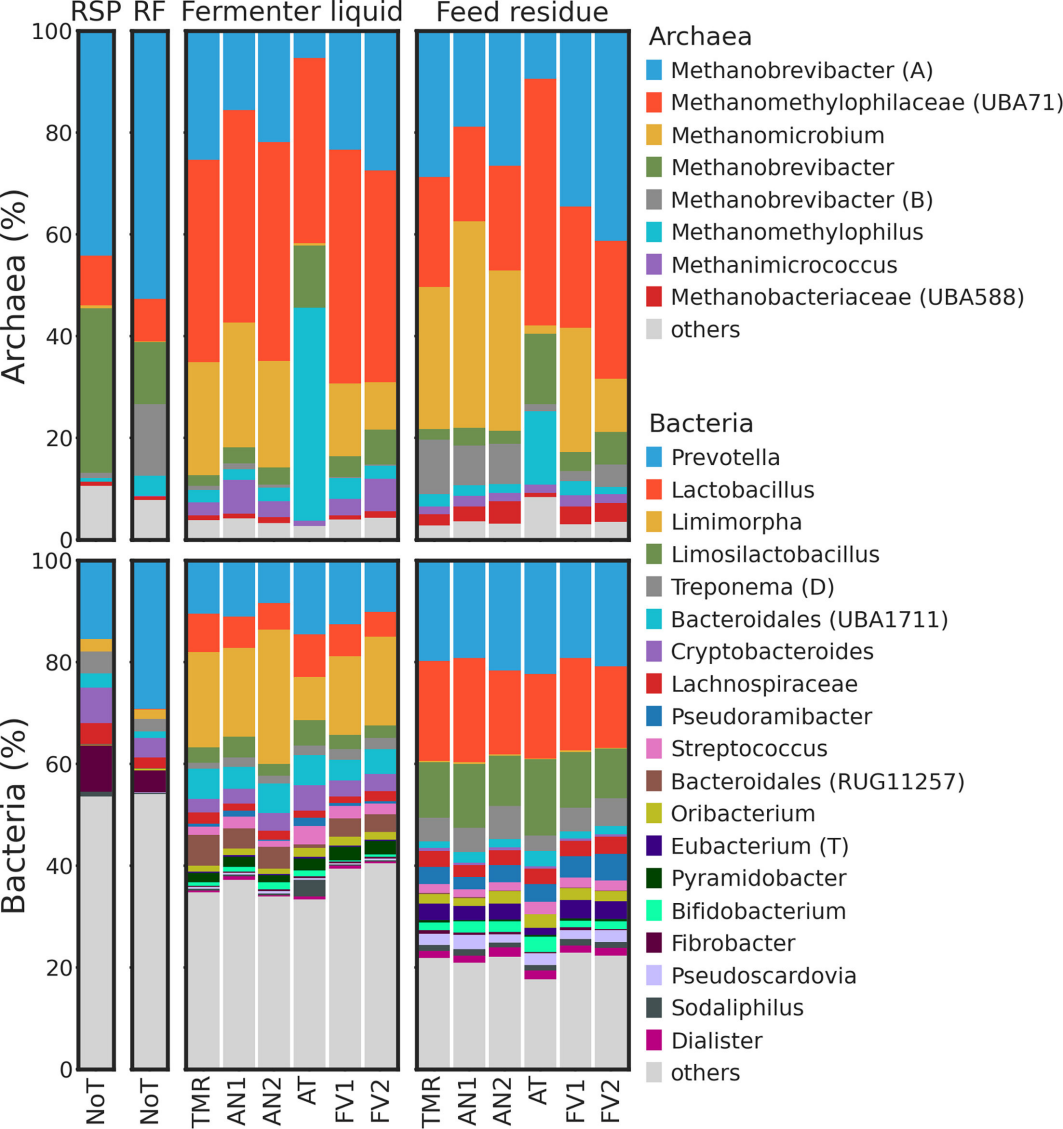
Average relative abundances of archaeal and bacterial genera. If sequences were not annotated the genus level, the last available taxonomy unit was indicated. Samples grouped by sample type and treatment. “NoT” stands for “no treatment.”

In original samples from the rumen that were used as starting material for the Rusitec, RSP, and RF, most of the archaeal reads were assigned to *Methanobrevibacter* A, *Methanobrevibacter*, and *Methanobrevibacter* B genera (Fig. 4). In the same sample types, relative abundances of *Methanobrevibacter* A and *Methanobrevibacter* were higher compared to FL and FR (all *P-adj* < 0.01). In FL dominance among archaeal genera shifted to the group UBA71 from *Methanomethylophilaceae*. In all Rusitec samples (FL and FR), relative abundances of group UBA71 from *Methanomethylophilaceae* and *Methanomicrobium* were higher compared to the RF and RSP (all *P-adj* ≤ 0.001). Supplementation of AT in the Rustitec samples resulted in the decrease of *Methanomicrobium* relative abundances (all *P-adj* ≤ 0.001). Among bacteria, *Prevotella* was the most abundant genus in RSP, RF, and FR samples, while in FL, dominance switched to *Limimorpha. Lactobacillus* and *Limosilactobacillus* were mostly represented in Rusitec samples, especially in FR.

#### Differentially abundant ASVs

**Fig 5:**
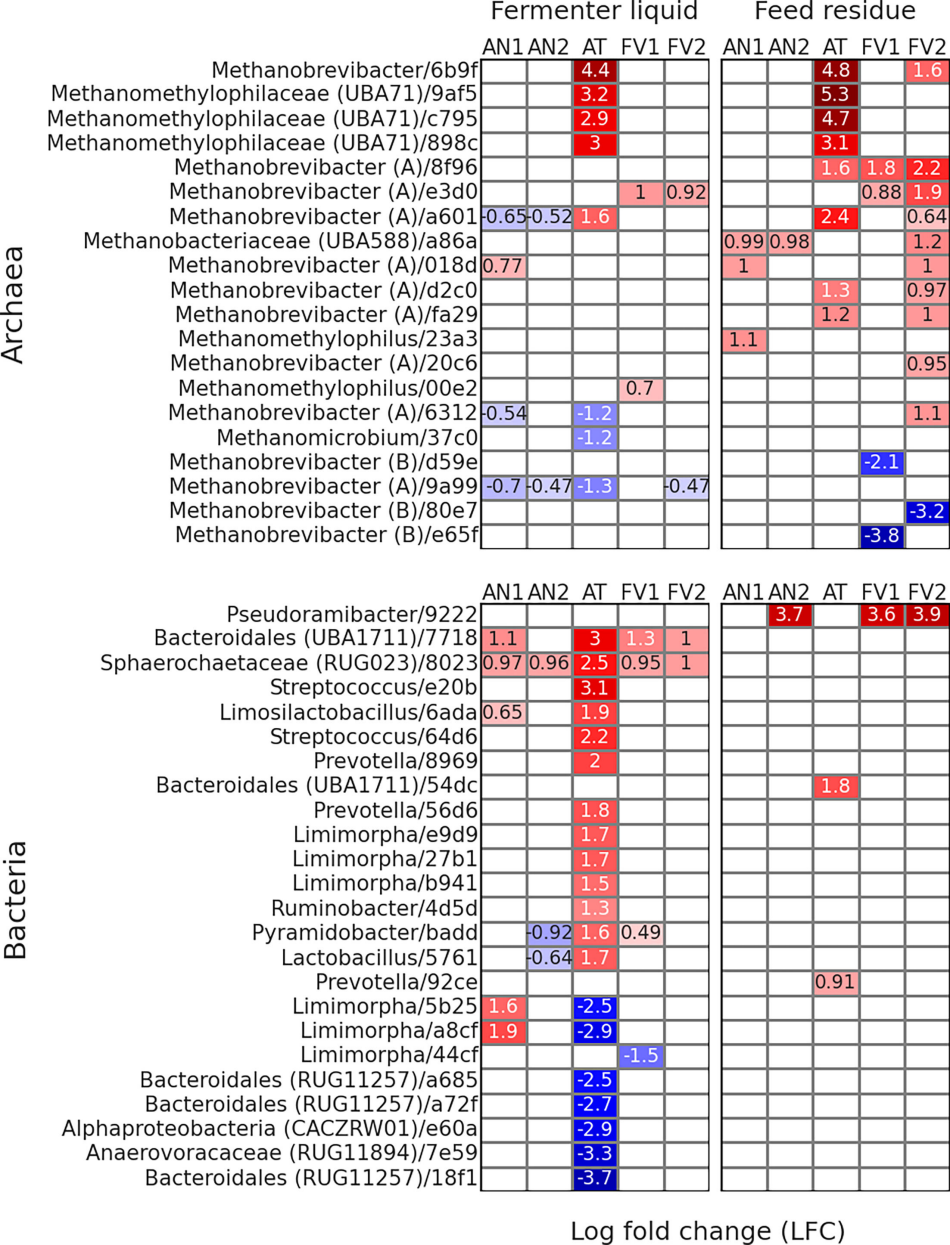
Differentially abundant archaeal and bacterial ASVs according to the ANCOM-BC. The formula included Treatment and Rusitec run as factors with TMR as treatment reference.

ASV abundances from all seaweed-supplemented treatments were compared to the TMR alone (Fig. 5) using ANCOM-BC. Since the analysis was performed at the ASV level, genus level annotation of the ASVs (if genus level was unavailable, then the last assigned taxonomy unit was used) was combined with four first characters from ASV id, separated by “/”.

The greatest number of differentially abundant ASVs between the treatment and the control was observed for AT-supplemented samples in both archaeal and bacterial communities. Archaeal ASVs *Methanobrevibacter*/6b9f, *Methanomethylophilaceae* (UBA71)/(9af5, c795, and 898c), and *Methanobrevibacter* (A)/a601 were elevated by AT supplementation in both FL and FR sample types. In addition, abundances of ASVs *Methanobrevibacter* (A)/(8f96, d2c0, and fa29) also were increased by AT in FR. In FL, AT treatment resulted in decreased abundances of *Methanobrevibacter* (A)/(6312 and 9a99) and *Methanomicrobium*/37c0. Among other treatments, AN1 resulted in higher abundances of *Methanobrevibacter* (A)/018d and lower levels of *Methanobrevibacter* (A)/(a601, 6312, and 9a99) in FL, while in FR ASVs, *Methanobacteriaceae* (UBA588)/a86a, *Methanobrevibacter* (A)/018d, and *Methanomethylophilus*/23a3 were decreased. AN2 decreased abundances of *Methanobrevibacter* (A)/(a601 and 9a99) in FL and increased the abundance of *Methanobacteriaceae* (UBA588)/a86a in FR. FV1 supplementation leveled up abundances of *Methanobrevibacter* (A)/a601 and *Methanomethylophilus*/00e2 in FL, while in FR, it increased abundances of *Methanobrevibacter* (A)/(8f96 and e3d0) and decreased *Methanobrevibacter* (B)/(d59e and e65f). Treatment FV2 mostly affected archaeal ASVs in FR. So, in FL, *Methanobrevibacter* (A)/e3d0 was elevated, and ASV *Methanobrevibacter* (A)/9a99 was decreased. In FR, ASVs *Methanobrevibacter*/6b9f and *Methanobrevibacter* (A)/(8f96, e3d0, a601, a86a, 018d, d2c0, fa29, 20c6, and 6312) were increased when FV2 was applied and *Methanobrevibacter* (B)/80e7 decreased.

In the bacterial dataset, AT supplementation in FL resulted in increased abundances of ASVs *Bacteroidales* (UBA1711)/7718, *Sphaerochaetaceae* (RUG023)/8023, *Streptococcus*/(e20b and 64d6), *Limosilactobacillus*/6ada, *Prevotella*/(8969 and 56d6), *Limimorpha*/(e9d9, 27b1, and b941), *Ruminobacter*/4d5d, *Pyramidobacter*/(badd), and *Lactobacillus*/5761, while in FR, *Bacteroidales* (UBA1711)/54dc and *Prevotella*/92ce increased their abundances. AT decreased abundances of *Limimorpha*/(5b25 and a8cf), *Bacteroidales* (RUG11257)/(a685, a72f, and 18f1), *Alphaproteobacteria* (CACZRW01)/e60a, and *Anaerovoracaceae*/(RUG11894)/7e59. AN1 supplementation leveled up counts of *Bacteroidales* (UBA1711)/7718, *Sphaerochaetaceae* (RUG023)/8023, *Limosilactobacillus*/6ada, and *Limimorpha*/(5b25 and a8cf). AN2 increased abundances of *Sphaerochaetaceae* (RUG023)/8023 in FL and *Pseudoramibacter*/9222 in FR and decreased *Pyramidobacter*/(badd) and *Lactobacillus*/5761 in FL. Both treatments FV1 and FV2 elevated ASVs *Bacteroidales* (UBA1711)/7718 and *Sphaerochaetaceae* (RUG023)/8023 in FL and *Pseudoramibacter*/9222 in FR. FV1 supplementation in FL increased abundances of *Pyramidobacter*/badd and decreased those of *Limimorpha*/44cf.

### Metagenomics

After quality control and host DNA removal, clean reads were used for metagenomes and metagenome-assembled genomes (MAGs) assembly, taxonomy profiling, and obtaining KEGG Orthology (KO) functional annotations. In total, after bin clustering and dereplication, 287 MAGs were assembled. Out of them, 67 MAGs with high quality (completeness ≥ 90% and contamination ≤ 5%) were deposited to the European Nucleotide Archive (ENA) repository (PRJEB65852).

#### Differentially abundant species

**Fig 6:**
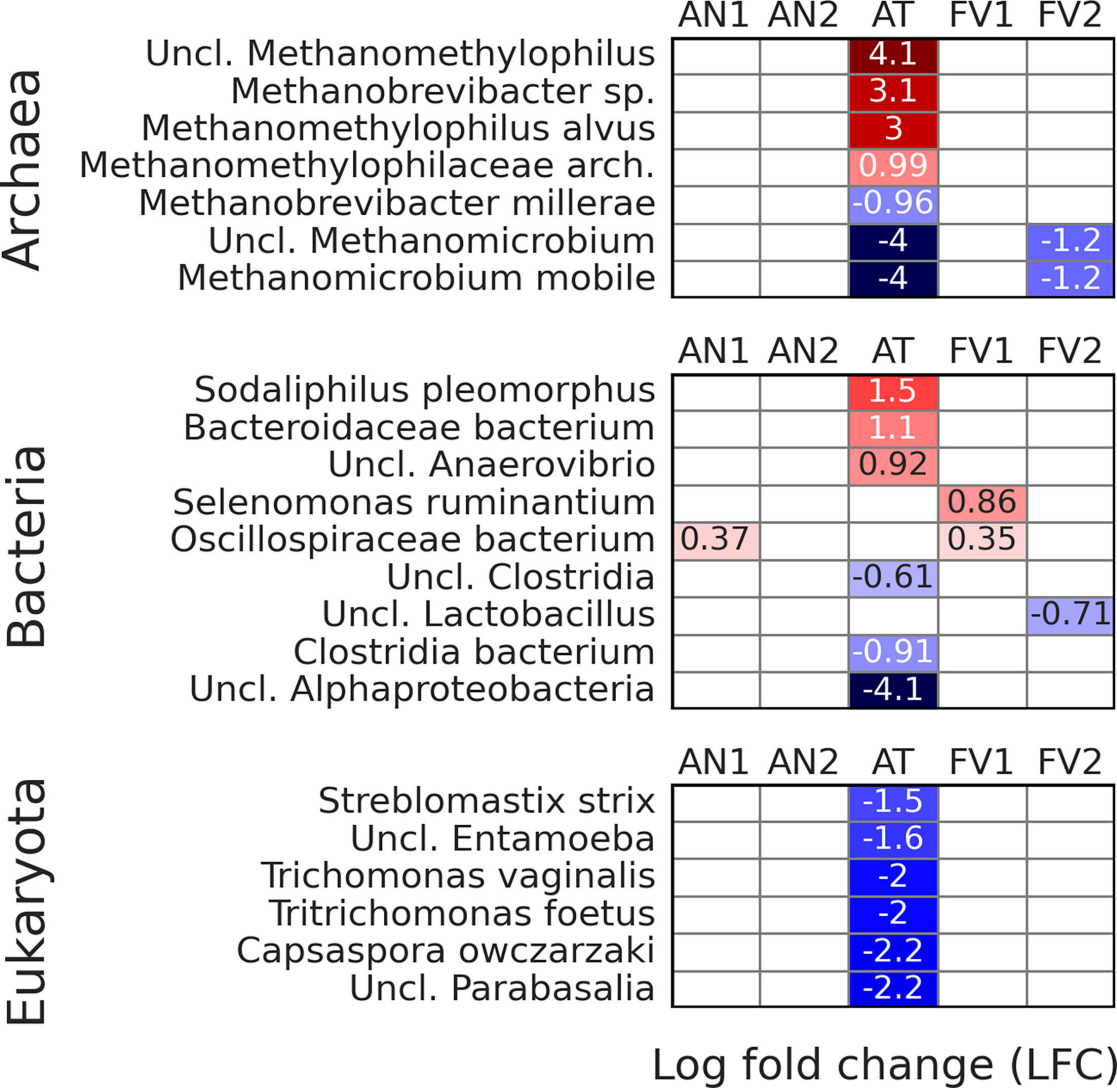
Differentially abundant archaeal, bacterial, and eukaryotic species according to the ANCOM-BC. The formula included Treatment and Rusitec run as factors with TMR as treatment reference.

Abundances of archaeal, bacterial, and eukaryotic species from seaweed-supplemented treatments were compared with TMR (Fig. 6).

Among archaea, some unclassified *Methanomethylophilus* species, *Methanobrevibacter* sp., *Methanomethylophilus alvus*, and Methanomethylophilaceae archaeon increased their abundances when AT was supplemented. The same treatment resulted in decreased counts of *Methanobrevibacter millerae*, unclassified *Methanomicrobium*, and *Methanomicrobium mobile.* Two latest archaeons were also suppressed by FV2 supplementation.

Regarding bacterial species, AT treatment increased the abundance of *Sodaliphilus pleomorphus* and two unclassified bacteria from *Bacteriodaceae* and *Anaerovibrio*. The growth of another two bacteria from *Clostridia* and one from *Alphaproteobacteria* was suppressed by the same treatment. Among other treatments, AN1 and FV1 increased the abundances of Oscillospiraceae bacterium, and FV1 also stimulated *Selenomonas ruminantium*, while FV2 decreased abundances of unclassified *Lactobacillus*.

Regarding Eukaryota, *Streblomastix strix*, *Trichomonas vaginalis*, *Tritichomonas foetus*, *Capsaspora owczarzaki*, and unclassified *Entamoeba* and *Parabasalia* were suppressed by AT supplementation.

#### Microbiota domains and Methanobrevibacter spp. clades ratios

**Fig 7:**
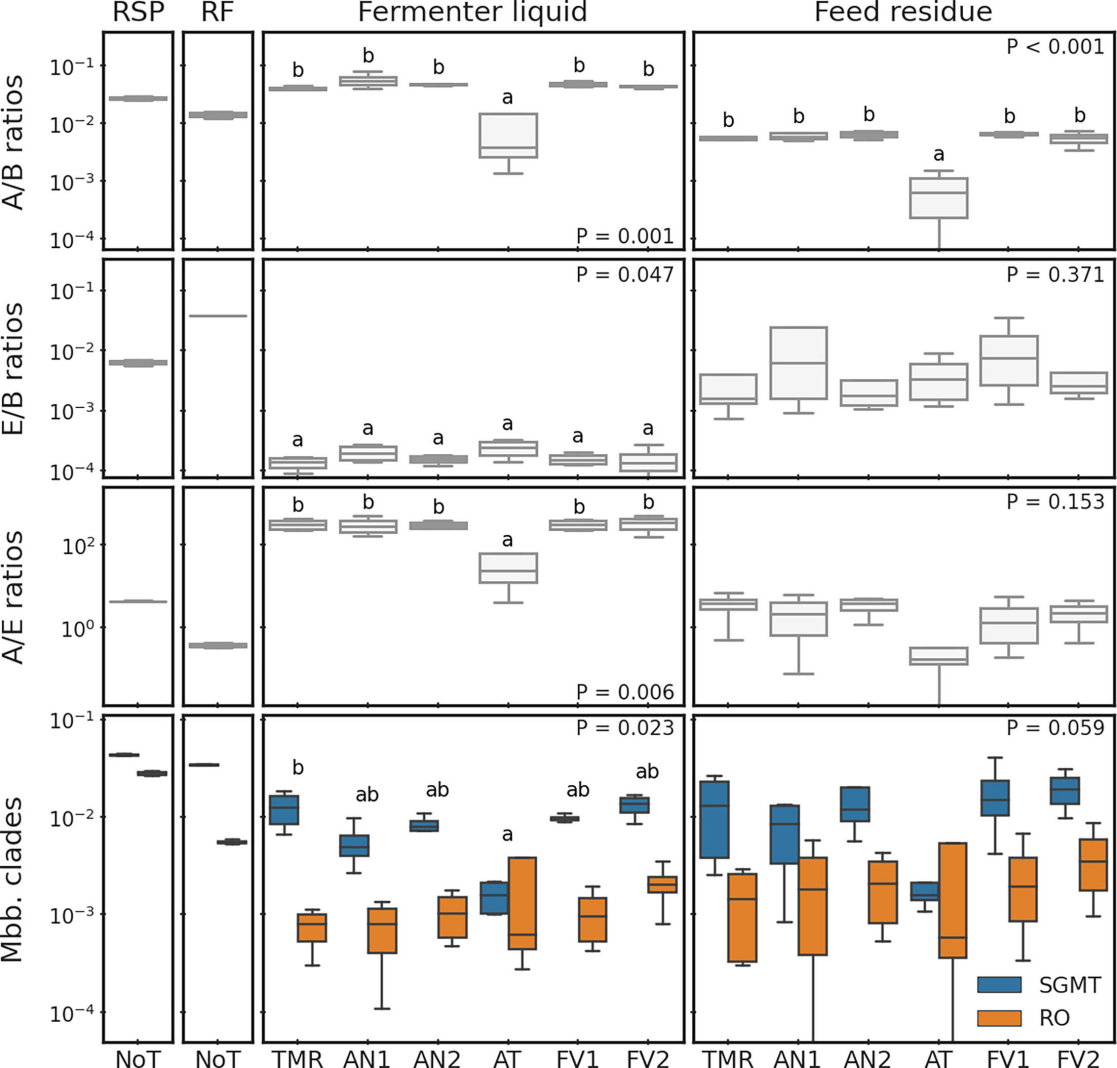
A/B, E/B, and A/E ratios and relative abundances of Methanobrevibacter clades. The Y-axis was plotted at the log scale of relative abundances. Clade “SGMT”: *Methanobrevibacter smithii*, *Methanobrevibacter gottschalkii*, *M. millerae*, and *Methanobrevibacter thaueri*. Clade “RO”: *Methanobrevibacter ruminatium* and *Methanobrevibacter olleyae*. In the upper or lower right area of each subplot with seaweed supplementations, *P* values of the ANOVA general test are plotted. Pairwise differences, based on *P*-adjusted values are denoted by the letters. For Mbb. clades subplots SGMT/RO ratios were tested. “NoT” stands for “no treatment.”

Archaea to Bacteria (A/B), Eukaryota to Bacteria (E/B), and Archaea to Eukaryota (A/E) ratios were affected by seaweed supplementation in FL (*P* = 0.001, 0.047, and 0.006, respectively), while in FR only E/B ratios were changed (*P* < 0.001; Fig. 7). Pairwise tests indicated that among A/B ratios of FL and FR and A/E ratios of FL, AT-supplemented samples had lower ratios compared to other treatments (all *P-adj* ≤ 0.012).

Besides domain ratios, seaweed supplementation affected ratios between *Methanobrevibacter* “SGMT” (*M. smithii*, *M. gottschalkii*, *M. millerae*, and *M. thaueri*) and “RO” (*M. ruminatium* and *M. olleyae*) clades in FL (*P* = 0.023), while in FR, only a trend was observed (*P* = 0.059; Fig. 7). When tested pairwise, in FL, among all pairs, AT had lower ratios compared to the TMR (*P-adj* = 0.017).

#### Differentially abundant functions

All obtained KO annotations were filtered and separated into two subsets: “Metabolism” and “Methane metabolism” (associated with CH_4_ metabolism).

**Fig 8:**
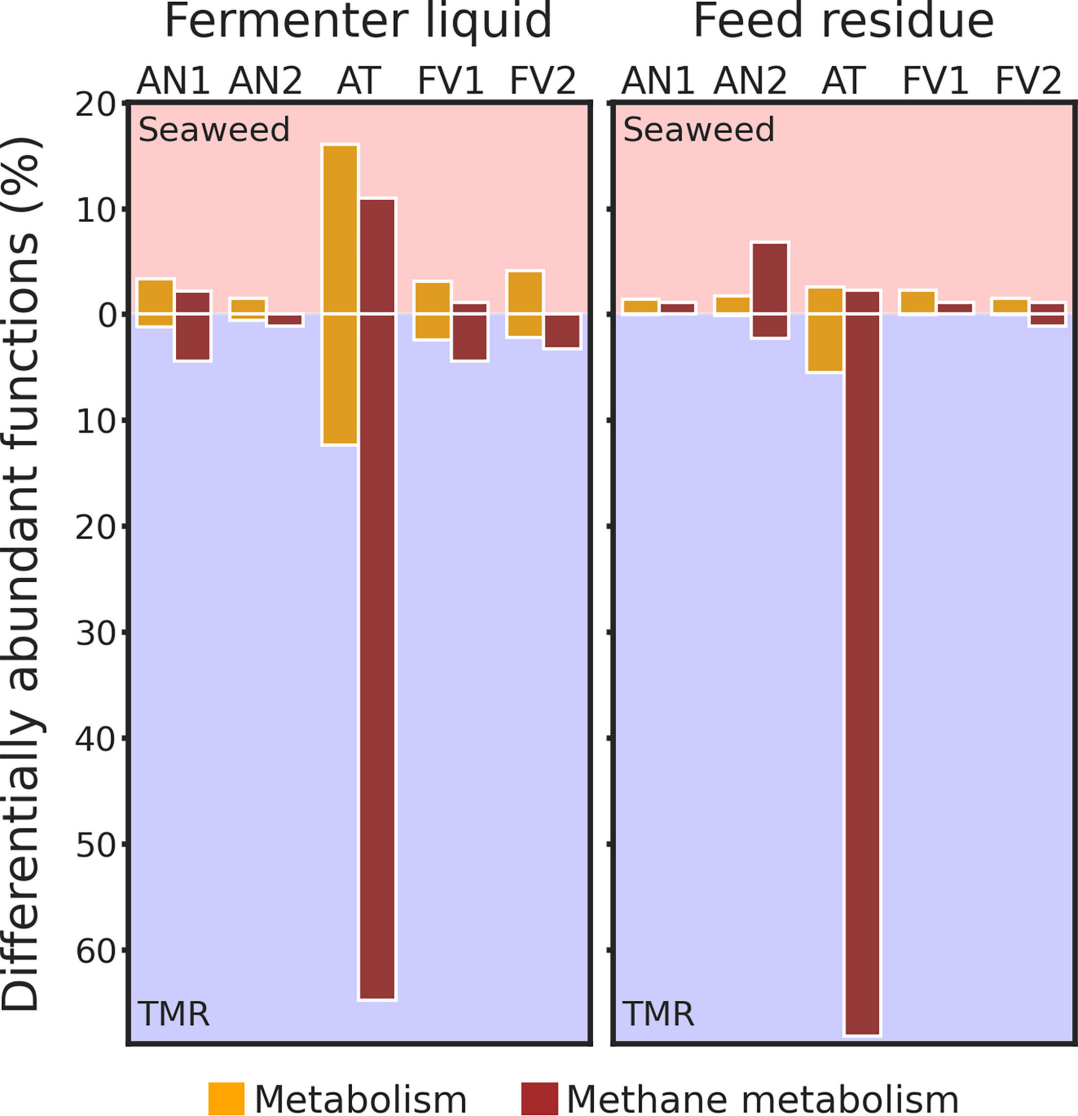
Percentage of differentially abundant functions (DAFs) between seaweed-supplemented treatments and TMR. Analysis was performed by MaAsLin2 for KO functions, subsampled to “Metabolism” and “Methane metabolism” categories. The analysis included Rusitec run as a random effect. Subsets are differentiated by the color. Red background was applied for DAFs, prevailed in seaweed-supplemented treatments and blue for more abundant in TMR. “NoT” stands for “no treatment.”

Among all seaweeds, *A. taxiformis* had the greatest effect on the microbial functional profiles (Fig. 8). From KEGGs of the “Methane metabolism” subset, abundances of more than 60% were decreased by AT. Its supplementation also demonstrated the highest percentage of increased KEGGs (around 10%) in FL.

**Fig 9:**
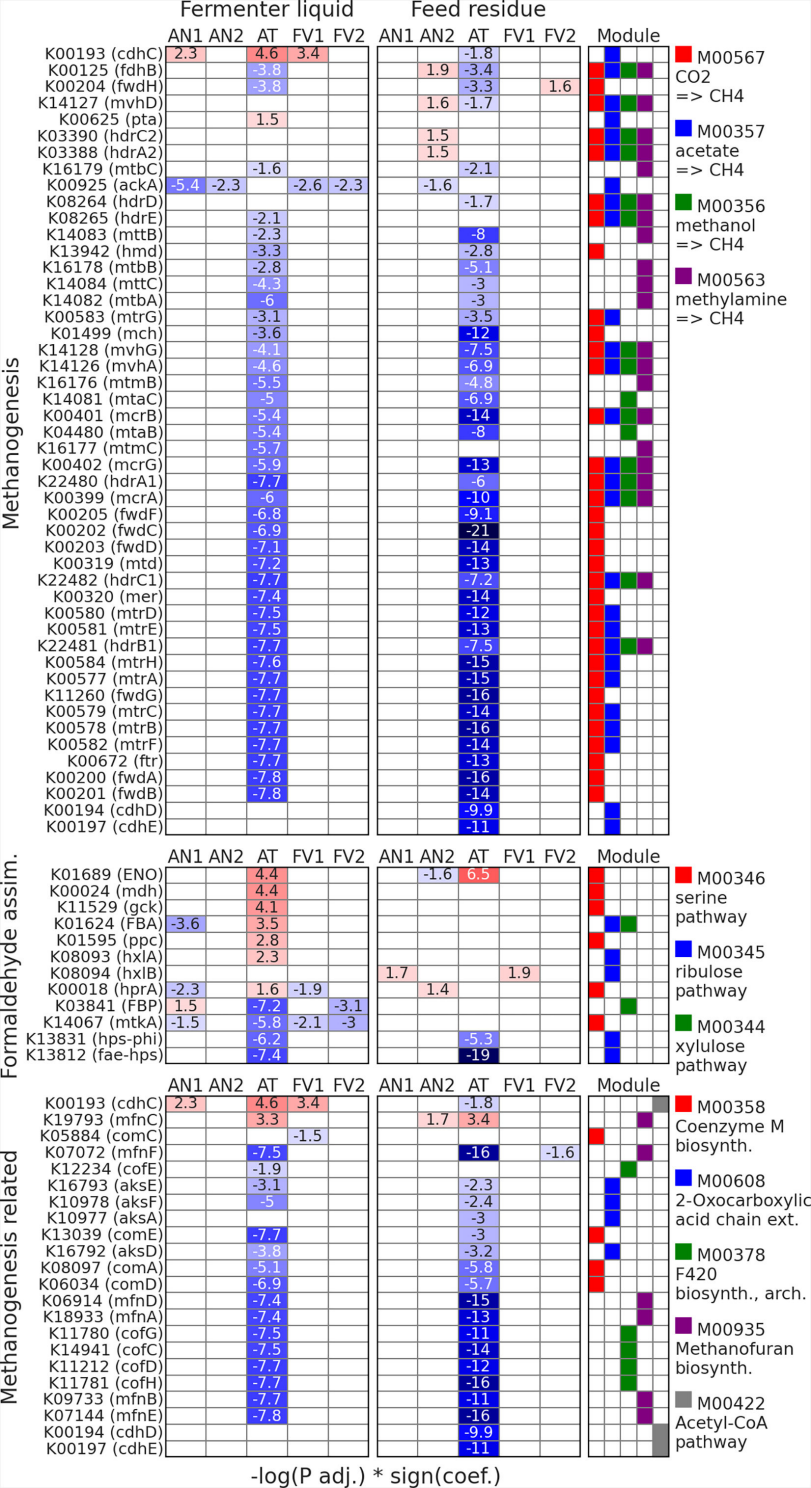
Differentially abundant KEGGs (MaAsLin2), from the “Methane metabolism” category. Analysis was performed for all treatments with TMR as the reference and Rusitec run as a random effect. The right panel indicates KEGG modules.

Differentially abundant functions (DAFs) from the “Methane metabolism” subset were plotted by the sample type, supplementation, and reaction module (Fig. 9). Among KEGGs that are directly involved in the CH_4_ production (marked as Methanogenesis), only two [K00193 (cdhC, acetyl-CoA decarbonylase/synthase) and K00625 (pta, phosphate acetyltransferase)] were augmented by AT. Abundances of all other DAFs from AT to TMR comparison that are firsthand involved in the methanogenesis were decreased in AT samples. Counts of KEGG [K00925 (ackA)] were negatively affected by all seaweeds except for AT. Several KEGGs [K00125 (fdhB), K14127 (mvhD), K03390 (hdrC2), and K03388 (hdrA2)] were leveled up by AN2 supplementation in FR samples.

The majority of the DAFs, involved in the CH_4_ utilization through formaldehyde assimilation, were positively affected by the AT supplementation and related to the serine pathway. Abundances of KEGGs from ribulose and xylulose pathways were mostly decreased when AT was supplemented.

Counts of related to 2-Oxocarboxylic acid KEGGs [K16793 (aksE), K10978 (aksF), K10977 (aksA), and K16792 (aksD)], cofactor F_420_ [K12234 (cofE), K11780 (cofG), K14941 (cofC), K11212 (cofD), and K11781 (cofH)], and MCR [K13039 (comE), K08097 (comA), and K06034 (comD)] biosynthesis was decreased in AT-supplemented samples. One KEGG K00193 (cdhC) from the Acetyl-CoA pathway was augmented in FL when AT was applied, while in FR, along with KEGGs K00194 (cdhD) and K00197 (cdhE) suppressed.

## DISCUSSION

### *In vitro* fermentation characteristics

Among all seaweed supplements tested, AT resulted in the largest decrease in CH_4_ concentration of total gas production in both HGT and Rusitec. These findings are in agreement with previous results (11–13, 28). Negative effects of AT supplementation on nutrient degradation (Table 1) were hardly observed, making this seaweed a desirable candidate as a feed supplement to mitigate methanogenesis. Only CP degradation was significantly reduced by AT supplementation. This indicates that a higher amount of CP was not degraded by microbes in the rumen and can therefore be used at the duodenum by the animal directly (29). Despite the great potential of AT in reducing CH_4_ production by ruminants, there are increasing concerns regarding the safety of such applications since the high content of its main anti-methanogenic compound—bromoform—has been reported to be toxic (30) and able to accumulate in milk (31). In addition, AT is inherently high in iodine concentration, and there are limits on how much iodine can be fed to animals producing meat and milk for human consumption in some countries (32). Therefore, further studies are required to investigate if AT inclusion can be reduced to levels that would avoid negative effects on animals and limit bromoform and iodine levels in the end products while minimizing methanogenesis.

In the Rusitec experiment, there was a high variation in fermentation traits and microbial data among the fermenters with AT supplementation, indicated by high variability in CH_4_ concentration compared to other seaweeds and distribution of FL samples in metataxonomics. This variability was also observed in the HGT experiment for the AT treatment, suggesting that either *A. taxiformis* itself or the heterogeneity of the applied stock material led to these changes. CH_4_ production was almost non-existent in three out of four fermenters, likely due to fermenter instability or inconsistent anti-methanogenic compounds in *A. taxiformis.*

The seaweeds sampled in Iceland (AN1 and FV1) had a non-significant reduction on CH_4_ concentration produced in the Rusitec (2.4% and 4.8%, respectively). However, the same seaweed species harvested at a similar time in Scotland (AN2 and FV2) did significantly decrease it (7.7% and 19%, respectively), especially FV2. Previous research on Icelandic AN and FV did show a reduction in CH_4_ concentration in total gas produced (reduction of 17% and 11%, respectively, at 5% seaweed inclusion) (15). The differences in whether or not these species reduce CH_4_ production are likely due to their bioactive content, such as concentrations of phlorotannins or total phenolic content. This extent of reduction in CH_4_ production for the AT harvested in the North Atlantic Ocean is consistent with previous findings with AT harvested in the Pacific Ocean which also generally showed a substantial reduction in CH_4_ production (12, 13, 28).

### Microbiome composition and domain ratios

Among all seaweeds applied, the most prominent effect on methane concentration was observed for AT supplementation (Fig. 1). The reducing effect of AT supplementation on CH_4_ production in ruminants was already reported in *in vitro* (12, 13, 33) and *in vivo* (11, 28) experiments. In our data, decreased methane production for AT-supplemented TMRs was accompanied by changes in archaeal counts, overall microbiome composition, and functional profiles.

Based on the “shotgun” metagenomic analysis, A/B ratios were lower in AT compared to all other supplementations and TMR alone in FL and FR samples (Fig. 7). This finding indicates that AT caused growth suppression of archaea and therefore the main rumen methanogens. Previous studies indicated that methanogen abundances are affected by various compounds, such as carbohydrates, lipids, peptides, phlorotannins, bromoform, and others, leading to a decline in methanogenesis (34, 35). Similar to our metagenome analysis, it was shown that AT supplementation resulted in drastically lower counts of archaeal methanogens in the Rusitec compared to the control, based on metataxonomics data (36). When A/B ratios were assessed based on our metataxonomics data, they were also lower in AT-treated samples compared to the TMR, AN1, and AN2 treatments in FL and compared to TMR and FV2 in FR (Fig. S2). Moreover, five samples from the 16S rRNA gene library with the lower counts of archaeal reads were attributed to the AT treatment (data not shown).

It is well known that archaeal methanogens are closely associated with protozoa (37, 38), and it was shown that abundances of protozoa are declining with time in the Rusitec (13). To test if the decline of the A/B ratio in AT-supplemented samples is associated with Eukaryota abundances, we also tested E/B and A/E ratios (Fig. 7). In the FL sample type, both ratios were significantly affected by seaweed supplementation. However, the E/B ratio demonstrated no differences between treatments when tested pairwise, while the A/E ratio was lower in AT-supplemented samples compared to the TMR alone and to all other treatments, suggesting that lower archaeal counts in the AT were not provoked by the decline in the total eukaryotic community. However, even if the total amount of eukaryotic microorganisms was not affected by the AT treatment, the abundance of some protozoa changed (Fig. 6). Thus, AT supplementation resulted in the decline of *S. strix*, *T. vaginalis*, *T. foetus*, and unclassified *Entamoeba*. Though it was not yet directly shown that *S. strix* produces hydrogen, several hydrogenases were identified in its single-cell metagenomics study (39). Both *T. vaginalis* and *T. foetus* are parasitic protists (40) and possess the ability to produce hydrogen due to the presence of special organelles—hydrogenosomes (41). *Entamoeba* species were shown to host similar to hydrogenosome organelles in their ability to produce hydrogen-mitosomes (42, 43). An important implication of these findings is that the decrease of the archaeal community representation under AT supplementation is associated with the decline of specific protozoa that are producing hydrogen, rather than with the overall decrease of Eukaryota. These results are consistent with a recent study that stated the decline in the methanogens activity was not solely dependent on the Rusitec-specific shifts in the microbiome composition but was due to AT supplementation, as it was the only treatment tested that caused it (36).

It has also been suggested that the composition of archaeal methanogens from the *Methanobrevibacter* genus, rather than their joined relative abundances, is responsible for methanogenesis inhibition (44). It was proposed that greater abundances of the *Methanobrevibacter* “SGMT” clade, which includes *M. smithii*, *M. gottschalkii*, *M. millerae*, and *M. thaueri*, and its ratio to another Mbb. clade “RO” (*M. ruminatium* and *M. olleyae*) are associated with higher production of CH_4_ (45–47). Our results are consistent with that hypothesis and demonstrated that “SGMT” to “RO” ratios were lower in AT-treated samples (Fig. 7) when compared to the TMR alone in FL. Though 16S rRNA gene amplicon sequencing approaches do not provide reliable species-level annotations, our results demonstrate that at the ASV level, numerous sequences assigned to the same genus, *Methanobrevibacter* (A), were separated into two clusters based on the positive or negative effect of the AT treatment at their abundances (Fig. 5). Both metataxonomics and metagenomics revealed the negative effects of AT treatment on *Methanomicrobium* abundances. In addition, based on the metagenomic data, the abundance of *M. millerae*, one of the “SGMT” clade members, decreased when AT was supplemented (Fig. 6). Of note, both FV1 and FV2 treatments increased abundances of *Methanobrevibacter* A and decreased *Methanobrevibacter* B. This indicated that the modulation of both methanogen abundances and their composition are important aspects in developing CH_4_ mitigation strategies.

Regarding bacterial genera, our data demonstrated that *Prevotella* abundances were positively affected by AT supplementation. It is likely that excessive availability of H_2_, accumulated due to suppressed overall methanogenesis, resulted in greater relative abundances of that genus members (Fig. 5), which are competing with methanogens for hydrogen utilization. *Prevotella* abundances were previously shown to be reversely associated with methane production (15, 35, 48). It was also recently shown that AT treatment caused an increase in *Prevotella* abundance in a Rusitec study (36). However, we should not completely exclude the possibility that hydrogen-consuming bacteria are somehow favored by AT treatment and decrease relative abundances of archaeal methanogens by direct competition. Some other bacteria that increased their abundances in AT-treated samples belonged to the *Streptococcus*, *Limosilactobacillus*, *Ruminobacter*, and *Limimorpha* genera. One of them, *Streptococcus*, is known as an anti-methanogenic bovicin component producer (49)*. Limosilactobacillus* member *Lactobacillus reuteri* inhibits methanogenesis (50). *Ruminobacter* is a genus of bacteria that produces formate, acetate, and succinate (51). Like *Methanobrevibacter* A methanogen, *Limimorpha* sequences were clustered into two groups of ASVs, positively or negatively affected by AT supplementation, though the exact reason for it is yet not known to us.

### Microbiome functional profiles

Among all seaweeds tested, *A. taxiformis* affected the most microbial functional profiles, especially functions associated with methanogenesis. The abundances of more than 60% of such functions were decreased after AT supplementation, while around 10% increased (Fig. 8). Such drastic effects corresponded with changes in *Methanobrevibacter* clades ratios and indicate that AT modulates methanogenesis not only through suppression of the total methanogens population but also via modulation of their taxonomical and functional profiles. For instance, among KEGGs that are directly involved in CH_4_ production, two from the acetoclastic pathway were augmented by AT, while the remaining DAFs, included in the hydrogenotrophic, methylotrophic, and acetoclastic methanogenesis pathways, decreased their abundances (Fig. 9, “Methanogenesis”). The reduction of methanogenesis can be accomplished not only by decreasing CH_4_ production but also by enhancing its utilization. In our study, we observed that most of the KEGGs, which were positively affected by the AT supplementation, were involved in the CH_4_ utilization through formaldehyde assimilation (Fig. 9, “Formaldehyde assim.”), especially involved in the serine pathway. Other important pathways that were affected by AT supplementation and that are related to the methanogenesis are 2-Oxocarboxylic chain extension, Acetyl-CoA pathway, and biosynthesis of such components as cofactor F_420_, coenzyme M (MCR), and methanofuran. It was shown that 2-oxocarboxylic acid is a precursor for coenzyme B and methanofuran biosynthesis, both of which participate in methanogenesis (52–54). In our study, KEGGs associated with 2-Oxocarboxylic chain extension (Fig. 9, “Methanogenesis related”) significantly decreased their abundances when AT was supplied. Acetyl-CoA is one of the intermediate products of acetoclastic methanogenesis (55) and one KEGG (cdhC) from its pathway was augmented in FL. Both cofactor F_420_ and coenzyme M (MCR) are crucial for hydrogenotrophic methanogenesis (56–58). In our analysis, KEGGs participating in the cofactor F_420_ and MCR biosynthesis were significantly reduced in AT-treated samples compared to TMR. Finally, methanofuran, as already mentioned, is an important component of methanogenesis (55). In our data, the abundance of one KEGG from its biosynthesis (mfnC) was increased by AT treatment, while the rest of the associated DAFs decreased their counts.

### Conclusions

The *in vitro* application of *A. taxiformis* as a feed supplement resulted in a drastic reduction of CH_4_ concentration produced with minor effects on nutrient degradation. The reduction was closer to 100% for three of the four replicates, indicating a significant potential for CH_4_ reduction. The present study suggests the AT mitigation of CH_4_ concentration is caused not only by the competitive inhibition of F_430_ coenzyme (18) but also through a decreased portion of the archaeal domain in the microbiome, as well as lower ratios of *Methanobrevibacter* “SGMT” to “RO” clades, and changes in the abundances of methane-associated microbial functions. Abundances of most of the KEGGs that are directly involved in methanogenesis were decreased, as well as KEGGs that are associated with it indirectly through the synthesis of methanogenesis-related compounds, such as F_420_ cofactor, coenzyme M, and methanofuran biosynthesis and extension of 2-Oxocarboxylic chain. Additionally, a small group of KEGGs that participate in CH_4_ assimilation via the serine pathway were more prevalent. *A. nodosum* and *F. vesiculosus* also decreased methane concentration in the total gas (2%–19%) at the 2.5% inclusion level; however, only the seaweed samples from Scotland decreased it significantly.

## MATERIALS AND METHODS

### Treatments

Five commercially available seaweeds were analyzed by inclusion in the TMR formulated for cattle and using different *in vitro* systems. The TMR for the HGT (TMR A) was composed of 20% corn grain, 20% soybean meal, 40% corn silage, and 20% grass silage. The TMR for the eHGT and the Rusitec (TMR B) was a mixture of 50% grass silage, 25% lupins, 15% soybean meal, and 10% wheat, intending a high crude protein content of 243 g/kg DM (Table 4) as required by the eHGT method. Seaweeds were harvested in Iceland [*A. nodosum* 1 (AN1), November 2019 and *F. vesiculosus* 1 (FV1), June 2019], Scotland [*A. nodosum* 2 (AN2), November 2019 and *F. vesiculosus* 2 (FV2), April 2018], and Portugal [*A. taxiformis* (AT), June 2019 at Faial Island]. Seaweeds were dried using natural energy sources at relatively low temperatures (around 40°C) and, together with the TMR components, were milled to pass a 1 mm screen. Seaweeds and TMR were analyzed according to official methods in Germany (59) for DM (method 3.1), CP (method 4.1.1), neutral detergent fiber on an ash-free basis after amylase pretreatment (aNDFom; method 6.5.1), acid detergent fiber on an ash-free basis (ADFom; method 6.5.2), crude ash (CA; method 8.1), and ether extract (EE; method 5.1.1). Starch was analyzed enzymatically according to Seifried et al. (60). The nutrient composition of seaweeds and TMR is shown in Table 4.

**TABLE 4:** Analyzed nutrient composition of the seaweeds and total mixed rations (g/kg DM)^*a*^

	CA	CP	CF	aNDFom	ADFom	EE	Starch^*b*^
AN1	220	59.2	35.9	226	377	19.0	n.d.
AN2	228	59.6	32.9	231	345	27.1	n.d.
AT	527	175	44.6	196	127	10.0	n.d.
FV1	230	120	36.9	230	340	15.5	n.d.
FV2	257	128	38.2	238	323	12.8	<LOQ
TMR A (HGT)	54.9	176	116	295	150	30.3	229
TMR B (eHGT + Rusitec)	73.1	243	145	295	190	49.4	58

^*a*^ AN, Ascophyllum nodosum; AT, Asparagopsis taxiformis; FV, Fucus vesiculosus; TMR, total mixed ration; HGT, Hohenheim Gas Test; eHGT, extended Hohenheim Gas Test; CA, Crude ash; CP, Crude protein; CF, Crude fibre; ADFom, Acid detergent fibre on ash free basis; aNDFom, Neutral detergent fiber on ash free basis and after amylase pretreatment; EE, Ether extract.

^*b*^ n.d., not detectable (< 3 g/kg DM); <LOQ, not quantifiable (< 3 – 6 g/kg DM).

### Hohenheim gas test

The five seaweeds were analyzed in the HGT in combination with TMR A to measure the total gas and CH_4_ concentration and calculate the ME concentration. Treatments were incubated according to Menke and Steingass (61) with small modifications for the CH_4_ measurements. An amount of 140 mg (CH_4_ production) or 200 mg (ME) was weighed into 100 mL graduated glass syringes, either TMR alone or a combination of 95% TMR and 5% seaweed on a DM basis. Each seaweed treatment was used with two repetitions, and the TMR treatment with three repetitions in four runs for the determination of ME and CH_4_ production. The syringes were sealed airtight with vaseline-greased plungers and prewarmed in an air-forced oven to 39°C. A reduced buffer solution was prewarmed in a water bath at 39°C under continuous flushing with CO_2_. Rumen fluid was collected from two Jersey cows. They were housed in groups and had *ad libitum* access to water, a TMR consisting of 33% corn silage, 33% grass silage, 23% hay, 10% barley straw, and 1% mineral mixture (by DM) and hay. Additionally, they were fed 4 kg of a concentrate consisting of 17% corn, 20% soybean meal, 25% barley, 28% wheat, 4% molasses, and 6% mineral mixture (by DM) per cow and day. Rumen fluid was collected prior to the morning feeding into prewarmed thermos flasks and then mixed, filtered through two layers of cheesecloth, and added to the buffer solution [1:2 (vol/vol)] under constant agitation. Thirty milliliter of buffered rumen fluid was dispensed into each prepared syringe. Afterward, they were put into a rotating disc in an air-forced oven at a constant temperature of 39°C. GP was recorded after 24 h and is accurate to ±0.5 mL. In the syringes for the CH_4_ detection, CH_4_ production was also analyzed after 24 h. For this purpose, syringes were connected to an infrared methane analyzer (PRONOVA Analysentechnik GmbH & Co., KG, Berlin, Germany) and calibrated using a reference gas (13.0 vol% CH_4_), and the produced gas was injected until the displayed methane production was constant.

In addition to the syringes incubated with treatments, four syringes with only buffered rumen fluid were used as blanks, and three syringes, each with concentrate or hay standard with known gas production were included in each run. Total gas and methane production were corrected using these blanks, and the GP with a correction factor was calculated with the GP of the standards.

The ME was calculated using the GP and the nutrient composition of the diets with the following equation by Boguhn et al. (62).

ME = 8.9695 + 0.04095 GP – 0.01267 CF + 0.04108 EE + 0.00387 CP + 0.00508 CA

where GP is in mL/200 mg and crude fiber (CF) and the other nutrient fractions in g/kg, all on a DM basis.

### Extended Hohenheim gas test

The eHGT method (63) with the modifications described by reference (64) was used to estimate “uCP at the duodenum” and RUP. Incubations were carried out similarly to the HGT described before with the following modifications: 130 mg DM of the treatments (100% TMR or 95% TMR and 5% seaweed on a DM basis) with and without the addition of 130 mg of a carbohydrate mixture (50% corn starch, 30% cellulose, and 20% sucrose) was weighed into syringes. Five subsequent runs were performed, and each run comprised an incubation over 8 h and an incubation over 24 h. Each incubation time contained one replicate of each treatment with and without the carbohydrate mixture. The gas production was recorded after 8 and 24 h of incubation, and syringes were immediately put on ice to stop further microbial fermentation. Steam distillation with subsequent titration (Vapodest 50, C. Gerhardt GmbH & Co. KG, Königswinter, Germany) was used to determine NH_3_-N in incubation residues. For this, 15 mL of phosphate buffer (90 g Na_2_ HPO_4_·12 H_2_O L^−1^, adjusted to pH 11.0 using sodium hydroxide) was added to the incubation residue, distilled NH_3_ was trapped in 3% boric acid, and titration was carried out with 0.05 M HCl. Concentrations of uCP and RUP were calculated as described by Wild et al. (65).

### Rusitec

#### Experimental design

Two consecutive runs in the rumen simulation system Rusitec were performed. The five seaweeds were analyzed together with TMR B, which also served as a control, at 2.5% inclusion level on a DM basis in exchange for TMR. Each run consisted of 7 days of adaptation period (d 0–6) and 7 days of sampling period (d 7–13). The setup of the Rusitec was described in detail by (15). In brief, 12 fermenters were arranged side by side, with six fermenters sharing one circulation thermostat (Lauda Eco E 4 S, Lauda-Königshofen, Germany) that kept the fermenters constantly at a temperature of 39°C and one buffer pump (Ismatec IPC ISM 931, Wertheim, Germany). The circulation thermostat was used as a blocking factor, and each treatment was randomly assigned to each block, resulting in four replications for each of the six treatments. The buffer solution was prepared according to McDougall (66) and continuously infused at a daily rate of 713 mL (75% of the fermenter capacity). Two nylon bags (120 × 70 mm, 100 µm pore size) containing 15 g of the respective treatments were put in a feed container doing vertical movement (10–12 strokes/min) and replaced by a new bag every 48 h. The removed bag was rinsed with a 50 mL buffer solution and squeezed moderately. From d 7–12, FRs were dried for 24 h at 65°C, weighed, pooled by fermenter, and milled to determine nutrient degradation. Each day before the feed bags were changed, the temperature, pH, and redox potential were measured in the FL (SenTix ORP, WTW Weilheim, Germany; BlueLine 14 pH IDS, SI Analytics, Mainz, Germany). Glass cylinders for the separation of gaseous and liquid effluent and bottles for the collection of liquid effluent (E) were placed in a 4°C tempered water bath. The effluent was weighed and sampled daily (70 mL/d) in the sampling period, pooled by fermenter, and stored at −20°C until it was centrifuged for 15 min at 24,000 × *g* to remove particles for the analysis of VFAs and NH_3_-N. Vacuum distillation and gas chromatography measurement (Hewlett-Packard 6890; Agilent, Waldbronn, Germany) were used to analyze VFA as described by Wischer et al. (2013). NH_3_-N was analyzed as described for the HGT. Gaseous effluent was measured daily in gas counters (BlueVCount, BlueSens gas sensor GmbH, Herten, Germany). CH_4_ concentration of total gas production was determined from gas-tight five-layered plastic-aluminum bags (Dr.-Ing. Ritter Apparatebau GmbH & Co. KG, Bochum, Germany) using an infrared methane analyzer (PRONOVA Analysentechnik GmbH & Co. KG, Berlin, Germany).

For the inoculation of the system, rumen content from three ruminal fistulated non-lactating Jersey cows was collected before the morning feeding. Cows were housed and fed as described for the HGT without the addition of concentrate. During the daytime, animals were allowed access to pasture. From each cow, 1 L RF was pumped from the liquid phase, 1 L squeezed out from the solid phase (RSP), and 200 g of squeezed solid phase was taken into prewarmed isolated containers. Afterward, rumen fluid was strained through two layers of cheesecloth and mixed at first in equal parts from the donor animals and then with a buffer solution (1:1). The mixture was stirred at 39°C and flushed with CO_2_ until the fermenters were filled. Solid rumen content was poured into nylon bags (60 g) and put together with a feed bag containing the respective treatment into the container of each fermenter. After 24 h, the feed bag with rumen content was removed and replaced by a treatment bag.

#### DNA libraries preparation

For metataxonomics and metagenomics microbiome analyses, samples were taken at d 0 from RSP (metataxonomics: 8; metagenomics: 2) and RF (metataxonomics: 8; metagenomics: 2), and at d 13 from E (metataxonomics: 24), FL (metataxonomics: 24; metagenomics: 24), and FR (metataxonomics: 48; metagenomics: 24). DNA extraction was performed with the FastDNA Spin Kit for soil (MP Biomedicals, LLC, Solon, OH, United States), according to the manufacturer’s instructions. DNA quantification was carried out with a NanoDrop 2000 (Thermo Fisher Scientific, Waltham, MA, United States). Extracted DNA was stored at −20°C.

For metataxonomics, bacterial (V1–V2 region) (67) and archaeal (Arch349-Arch806 primers) (9, 68) sequencing libraries were prepared. Targeted 16S rRNA gene regions were amplified in two PCR steps, one for each of the primers. Barcodes (6-nt) and linker (2-nt) were attached to the forward primer. Reverse primer contained sequences specific to multiplex and index primers. The resulting amplicons were normalized by the SequalPrep Normalization Kit (Invitrogen Inc., Carlsbad, CA, United States) and sequenced with the 250 bp paired-end Illumina NovaSeq 6000 platform. Metagenomics samples were sequenced with 150 bp paired-end in an Illumina NovaSeq 6000.

### Statistical analyses and bioinformatics

Statistical analysis of the HGT was done with the MIXED procedure of SAS 9.4 for Windows (SAS Institute Inc., Cary, NC) using a one-way ANOVA with the seaweed as a fixed effect and the run and syringe position as random effects. For the eHGT, effective uCP and effective RUP were estimated for assumed ruminal passage rates of 2%/h, 5%/h, and 8%/h by plotting uCP and RUP values (y) against the natural logarithm of the incubation time (x) in a linear regression model using PROC MIXED of SAS. The gas data, nutrient degradation, NH_3_-N, and VFA observed in the Rusitec were analyzed with a one-way ANOVA in SAS using the mixed procedure. The treatment was the fixed effect, and run, circulation thermostat, fermenter, and day were used as random effects. When treatment differences were identified in an ANOVA, a multiple *t* test [Fisher’s least significant difference (LSD) test] was used to distinguish between treatments. All residuals were checked graphically for the normal distribution and homogeneity of variance.

For metataxonomics, raw reads were demultiplexed with Sabre (https://github.com/najoshi/sabre) and analyzed in Qiime2 (v2023.5) (69). Primer removal was performed by the q2-cutadapt (70). Quality filtering, error correction, dereplication, and pair merging were accomplished by the q2-dada2 (71). Resulted ASVs were classified by VSEARCH-based consensus (72) and pre-fitted sklearn-based classifiers (73) against the GTDB database (v. 214.1) (74). Reference reads were obtained and processed by RESCRIPt (75). Alpha diversity was assessed by Faith’s PD (76) and beta diversity by Bray-Curtis (77) distances. Alpha diversity metrics and relative abundances of the most abundant taxa were tested with the ANOVA (78) and beta diversity distances with Adonis (999 permutations) (79). Due to the high similarity between E and FL sample types, they were pooled as technical replicates and referred to as FL. In both cases, tests were performed separately within FL and FR sample types with the formula “Treatment + Rusitec run”. *P*-values from diversity pairwise tests were adjusted using the Benjamini-Hochberg procedure (80). Differentially abundant ASVs were detected by ANCOM-BC (81), with the formula “Treatment + Rusitec run” and performed on the sequences with relative abundance ≥1% and prevalence ≥20%. Statistical analysis of relative abundance dynamics of specific microbial genera was performed with the Kruskal-Wallis test (82).

For metagenomics, raw reads were quality controlled and cleaned of the host DNA using “ReadQC” module from the MetaWrap (83). Metagenome co-assemblies were created with metaSpades (84). Function annotation was performed by the SqueezeMeta pipeline (v1.4.0) (85). RNAs and open reading frames (ORFs) were predicted with Barrnap (86), Aragorn (87), and Prodigal (88). Taxonomy classification of 16S rRNA gene sequences from metagenomes was performed using the Ribosomal Database Project (RDP) classifier (89). Domain and *Methanobrevibacter* clades ratios were tested within sample types by ANOVA (78) with the formula “Treatment + Rusitec run.” A similarity search for the KO (90) database was implemented by Diamond (91). Reads were mapped to the contigs by Bowtie2 (92). DAFs between treatments were detected by MaAsLin2 (93) with TMR as a reference and Rusitec run as a random factor. Bins were assembled by sample types with MaxBin2 (94) and Metabat2 (95) and then combined by DAS Tool (96). Bins from different sample types were pooled, clustered (95%), and dereplicated to MAGs by mOTUlizer (97) and SuperPang (98). Taxonomy annotation of MAGs was carried out by RDP classifier (89) and GTDB (v. 214.1) (74) following manual curation.

## Data Availability

The data sets generated and/or analyzed during the current study are available in the PRJEB65852 repository. The code used for bioinformatics can be found at https://github.com/timyerg/Rusitec_2020.

## SUPPLEMENTAL MATERIAL

Supplemental figures spectrum.03942-23-s0001.pdf Fig. S1 and S2.

Supplemental tables spectrum.03942-23-s0002.xlsx Tables S1 and S2.

ASM does not own the copyrights to Supplemental Material that may be linked to, or accessed through, an article. The authors have granted ASM a non-exclusive, world-wide license to publish the Supplemental Material files. Please contact the corresponding author directly for reuse.
