# Supplementary material for: The effect of Asparagopsis taxiformis, Ascophyllum nodosum, and Fucus vesiculosus on ruminal methanogenesis and metagenomic functional profiles in vitro
Source: Microbiol Spectr. 2024 Sep 30;12(11):e03942-23. doi: 10.1128/spectrum.03942-23 (PMC11542596; doi:10.1128/spectrum.03942-23)
Supplement: Supplemental figures — Fig. S1 and S2. [file spectrum.03942-23-s0001.pdf]

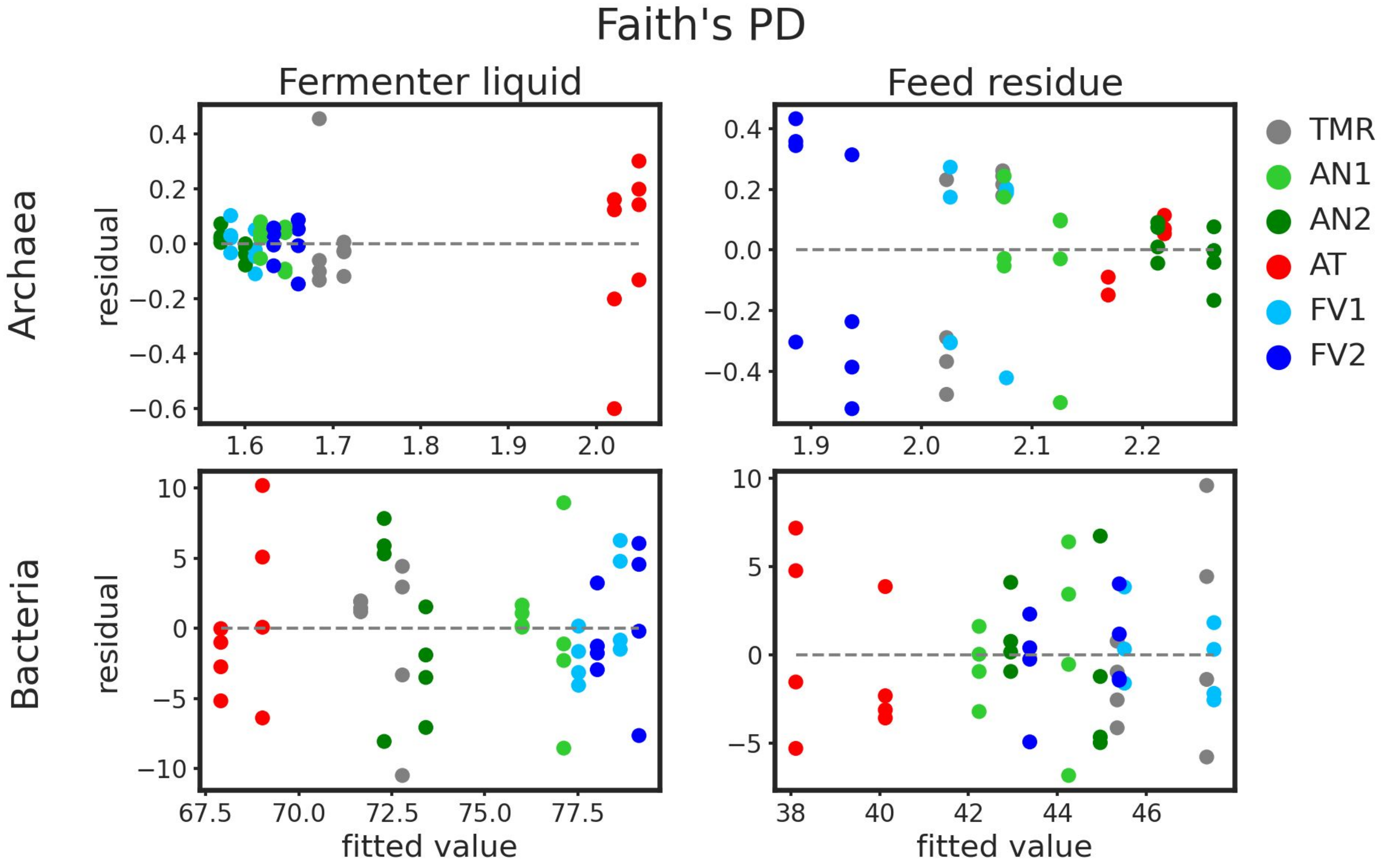


**Figure S1.** ANOVA residual vs fitted value plots for the effect of seaweed supplementation on archaeal and bacterial Faith's phylogenetic diversity.

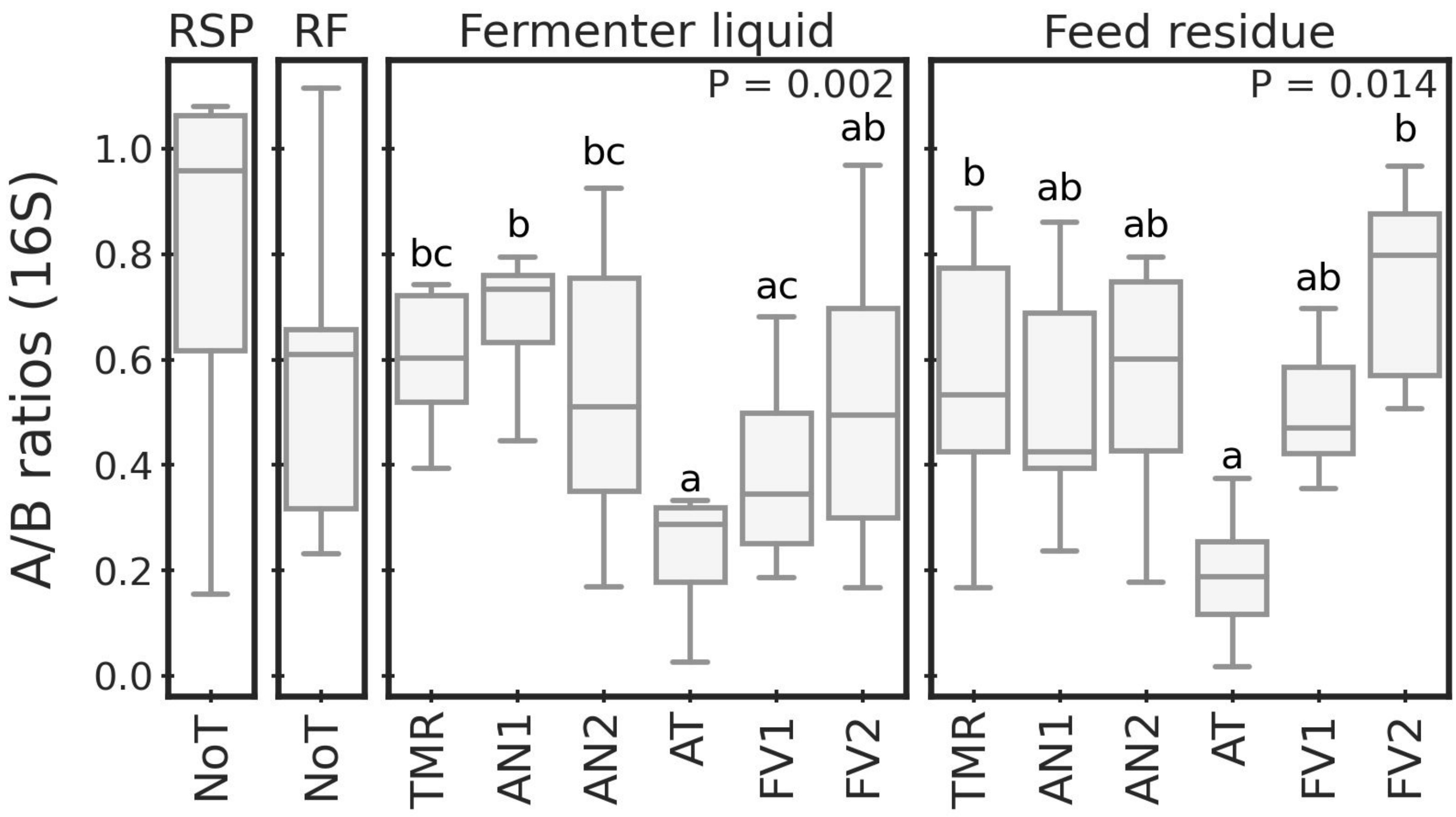


**Figure S2.** Archaea to bacteria ratios based on 16S data absolute abundances*.* “NoT” stands for “no treatment”.
